# Clinical correlation and prognostic significance of immunofluorescence in renal biopsies of patients having Glomerulonephritis

**DOI:** 10.12669/pjms.37.1.2497

**Published:** 2021

**Authors:** Fauzia Zafar, M. Faisal Mehar, Afsheen Asghar Khan, Rabia Saleem Safdar

**Affiliations:** 1 Fauzia Zafar, FCPS (Pediatric Medicine). Department of Pediatrics, Ward Number 19, Nishtar Medical University Hospital, Multan, Pakistan; 2 M. Faisal Mehar, FCPS (Pediatric Medicine). Department of Pediatrics, Ward Number 19, Nishtar Medical University Hospital, Multan, Pakistan; 3 Afsheen Asghar Khan, FCPS (Pediatric Medicine). Department of Pediatrics, Ward Number 19, Nishtar Medical University Hospital, Multan, Pakistan; 4 Rabia Saleem Safdar, FCPS (Pediatric Medicine). Department of Pediatrics, Ward Number 19, Nishtar Medical University Hospital, Multan, Pakistan

**Keywords:** Glomerulonephritis, immunofluorescence, renal biopsy

## Abstract

**Objective:**

To correlate the immunofluorescence (IF) findings on renal biopsies of patients of glomerulonephritis (GN) with the clinical course of the disease.

**Methods:**

This retrospective descriptive study was done at the Department of Pediatrics Medicine Unit-I, Nishtar Hospital Multan, from January 2008 to January 2019. A total of 387 cases of both gender, aged up to 16 years, diagnosed having GN on the basis of renal biopsies by light microscopy (LM) and IF findings, were included. Outcome as remission, partial remission, no remission with stable kidney disease, no remission with progressive kidney disease and end-stage kidney disease (ESKD) were computed. Chi square test was applied to see the correlation of IF findings and outcome by taking p value less than 0.05 as statistically significant.

**Results:**

Focal segmental glomerulosclerosis (FSGS) was found to be the commonest histopathology finding noted in 158 (40.8%) followed by mesangioproliferative GN 74 (19.1%) and membranous nephropathy 42 (10.9%). Complete remission was observed in 145 (37.5%) cases whereas ESKD was seen in 26 (6.7%). Distinct pattern of IF findings were shown when distribution of IF findings were seen with respect to all study variables (p value < 0.001). For outcome, 134 (51.3%) IF negative cases had complete remission while 93 (35.6%) negative IF findings also had partial remission. ESKD was seen among 14 (25.9%) IgM positive and three (33.3%) IgA positive cases.

**Conclusion:**

Immunofluorescence proved an important diagnostic tool in reaching the exact diagnosis in various types of GN. Distinct correlation between IF findings and clinical course of various types of GN was observed. IF negative cases had better outcome and was not having progressive course of disease so prognosis remained better than IF positive cases in this study.

## INTRODUCTION

Glomerulonephritis (GN) is a range of conditions that can affect kidneys specifically the glomeruli.^1^ All types of GN start as a unique initiating stimulus while inflammation and fibrotic events progress into final pathway of progressive renal damage.^2^ These present as nephrotic syndrome, nephritic syndrome, non-nephrotic proteinuria, isolated hematuria or acute/chronic renal failure. GN could be acute or chronic in terms of onset. Poststreptococcal GN is the best example of acute glomerulonephritis (AGN).^3^

Corticosteroids are the first line treatment. Majority of children respond well to this treatment. However, there is a subset of children who do not respond to this therapy and they are said to have steroid resistant nephrotic syndrome (SRNS). In such cases renal biopsy is indicated and underlying cause may be FSGS, MesPGN, membranous glomerulonephritis, MPGN, IgA nephropathy or C3 glomerulopathy.^4^-^6^

Renal biopsy being an invasive procedure with possible risk of complications, needs to be supported by all methodologies like light microscopy (LM), electron microscopy (EM) and Immunofluorescence (IF), but requires strict clinico-pathological correlation.^4^,^5^ In the diagnosis of GN, the direct IF has become a necessary morphological tool for proving immunological mechanisms involved.^7^ IF is a process of labeling of antibodies and antigens with fluorescent dyes especially for the purpose of demonstrating a particular antigen or antibody in a tissue preparation or smear. Whatever data exists, researchers endorse that IF along with LM needs to be done for the accurate diagnosis of GN. Cases of lupus nephritis, IgA nephropathy and IgM nephropathy are very difficult to diagnose with LM only so IF can provide an additional tool for the exact diagnosis and estimation of the clinical course in these conditions as treatment modalities in these conditions require aggressive approach influencing the progression.^7^ Buch AC et al found that IF helped in accurate diagnosis of 12% cases of IgA nephropathy and 5.3% cases of lupus nephritis.^8^

Direct immunofluorescence microscopy and correlation with LM, clinical, biochemical and serological markers should be done on a regular basis for the correct diagnosis of glomerular diseases. It is necessary to determine the exact type and pattern of kidney injury, so we can make proper management plans to minimize the glomerular injury and hence expect the good prognosis.

In Pakistan it is not a usual practice to perform IF and EM in all renal biopsies. The objective of the study was to correlate the immunofluorescence findings on renal biopsies of patients of glomerulonephritis with the clinical course of the disease. This study will help in demonstrating the relationship of IF positive GN with the clinical course and eventually the outcome of the glomerular disease.

## METHODS

This retrospective descriptive study was done at the Department of Pediatrics Medicine Unit-I, Nishtar Hospital Multan, from January 2008 to January 2019. A total of 387 cases of both gender, aged up to 16 years, diagnosed having GN on the basis of renal biopsies by LM and IF findings, were included. All the cases other than GN on renal biopsies, age less than six months and all those having GN who left against medical advice or lost their follow up were not included. Approval from ethical committee of the institution was taken for this study (Ref. No. 4967-90, Dated: 03-03-2020).

Patient’s demographic along with clinical and laboratory data, urine dipstick for proteinuria, renal functions, 24 hours urinary protein and ultrasonography findings of kidneys, ureters and bladder (KUB) were noted. Findings were collected from case records. Children were followed for the period of 12 months, if remained constant during this period, the outcome was documented as follows: Complete Remission was labeled as proteinuria <4 mg/m^2^/hour or nill to trace on urine dipstick testing for three consecutive days. Partial Remission was taken as proteinuria >4 mg/ m^2^ /hour but < 40 mg/ m^2^ /hour or 1+ to 2+ protein on urine dipstick testing for three consecutive days. No remission (Relapse) was labeled as proteinuria >40 mg /m^2^ /hour or protein:creatinine ratio >2 or ≥3+protein on urine dipstick testing for three consecutive days. No remission with stable kidney disease was considered as proteinuria >40 mg /m^2^ /hour or protein:creatinine ratio >2 or ≥3+ protein on urine dipstick testing for 3 consecutive days along with normal renal parameters. No remission with progression of kidney disease was proteinuria >40 mg /m^2^ /hour or protein:creatinine ratio >2 or ≥3+ protein on urine dipstick testing for three consecutive days along with deranged renal parameters. End stage kidney disease was marked as chronic kidney disease with stage v (GFR <15 mg/minute/1.73m^2^).

SPSS (Statistical Package for Social Science USA) version 24.0 was used for data entry and analysis. Frequency and percentages were calculated for qualitative variables like IF findings, histopathological findings and outcome while mean and standard deviation were calculated for age. Chi square test was applied to see the correlation of IF findings and outcome by taking p value less than 0.05 as statistically significant.

## RESULTS

Out of a total of 387 patients, 201 (51.9%) were male and aged above 10 years (n=227, 58.7%). Overall, mean age was found to be 8.65±3.26 years. Distribution of number of GN cases with regards to IF finding is shown in Figure-I. IF findings were negative in 261 (67.4%) while IgM was found positive in 54 (14.0%) followed by IgG positive 36 (9.3%). FSGS was found to be the commonest histopathology finding noted in 158 (40.8%) followed by Mesangioproliferative GN 74 (19.1%) and Membranous nephropathy 42 (10.9%). Outcome of the study participants showed complete remission was observed in 145 (37.5%), Partial remission in 121 (31.3%), No remission with progression disease in 40 (10.3%) cases, No remission with stable disease in 55 (14.2%) cases and ESKD was seen in 26 (6.7%).

**Fig.1:**
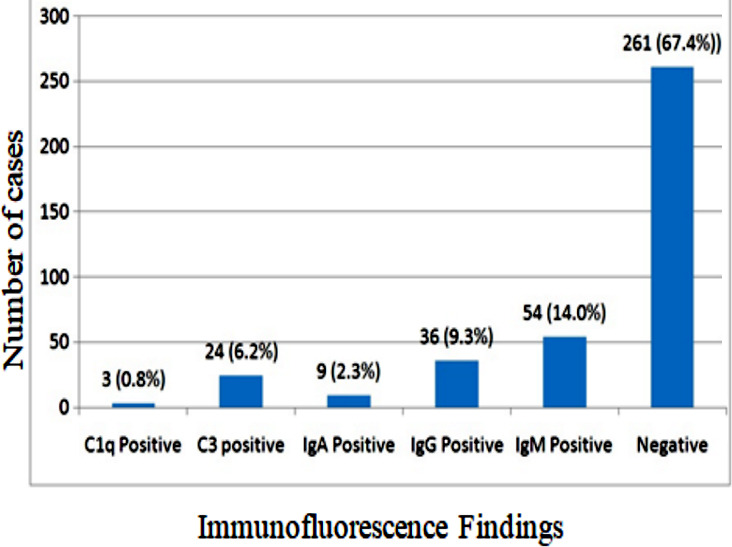
Distribution of GN cases with regards to Immunofluorescence Findings in children undergoing renal biopsies (n=387).

Distinct pattern of IF findings were found when distribution of IF findings were seen with respect to all study variables (p value < 0.001). Table-I. It was observed that C3, IgG and IgM positive (100%, 67.4% and 83.3% respectively) children were significantly older (> 10 years) compared to negative cases (p-value <0.001). It was also noted that C1q, IgA and IgM positive cases (100%, 88.9% and 70.4% respectively) were significantly dominated by male gender (p-value < 0.001). In terms of histopathology, all 3 (100%) C1q positive cases had diffuse proliferative GN, 18 (75.0%) C3 positive cases had lupus nephritis, 6 (66.7%) IgA positive cases were having diffuse proliferative GN, 29 (53.7%) IgM positive cases had FSGS while 114 (43.7%) FSGS and 65 (24.9%) MesPGN cases had negative IF findings. For outcome, 134 (51.3%) IF negative cases had complete remission while 93 (35.6%) negative IF findings also had partial remission. ESKD was seen among 14 (25.9%) IgM positive and 3 (33.3%) IgA positive cases.

**Table-I:** Relation of IF findings with study variables.

*Study Variables*		*IF Findings*						

		C1q Positive	C3 Positive	IgA Positive	IgG Positive	IgM Positive	Negative	P Value
Age (years)	<10	3 (100%)	0	5 (55.6%)	11 (30.6%)	9 (16.7%)	132 (50.6%)	<0.001
	>10	0	24 (100%)	4 (44.4%)	25 (67.4%)	45 (83.3%)	129 (49.4%)	
Gender	Female	0	19 (79.2%)	1 (11.1%)	17 (47.2%)	16 (29.6%)	133 (51.0%)	<0.001
	Male	3 (100%)	5 (20.8%)	8 (88.9%)	19 (52.8%)	38 (70.4%)	128 (49.0%)	
Histopathology	DPGN	3 (100%)	0	6 (66.7%)	0	0	9 (3.4%)	<0.001
	FSGS	0	0	0	15 (41.7%)	29 (53.7%)	114 (43.7%)	
	Lupus Nephritis	0	18 (75.0%)	0	0	0	9 (3.4%)	
	MCD	0	0	0	0	0	49 (18.8%)	
	MN	0	3 (12.5%)	0	18 (50.0%)	8 (14.8%)	13 (5.0%)	
	MesPGN	0	0	3 (33.3%)	0	6 (11.1%)	65 (24.9%)	
	MPGN	0	3 (12.5%)	0	3 (8.3%)	11 (20.4%)	2 (0.8%)	
Outcome	Complete Remission	0	3 (12.5%)	0	5 (13.9%)	3 (5.6%)	134 (51.3%)	<0.001
	No Remission, SKD	2 (66.7%)	3 (12.5%)	2 (22.2%)	7 (19.4%)	20 (37.0%)	21 (8.0%)	
	No Remission, PKD	1 (33.3%)	6 (25.0%)	3 (33.3%)	9 (25.0%)	13 (24.1%)	8 (3.1%)	
	Partial Remission	0	9 (37.5%)	1 (11.1%)	14 (38.9%)	4 (7.4%)	93 (35.6%)	
	ESKD	0	3 (12.5%)	3 (33.3%)	1 (2.8%)	14 (25.9%)	5 (1.9%)	

## DISCUSSION

In the present study, 67.4% of the cases had negative LF findings. We also noted that most of the cases with immunoflouresce negative had better outcome. Immunoflouresce positivity shows severe disease and poor outcome too. Researchers have shown that LM only provides morphological patterns of GN however to reach the exact diagnosis of GN disease, correlation between clinical data, LM as well as IF findings are crucial.^9^ In our institution, IF when combined with LM, gave accurate results in most cases despite the lack of availability of EM.

Immune mechanisms are responsible for glomerular injury in most cases of glomerulonephritis. In the diagnosis of GN, the direct immunofluorescence (IF) has become a necessary morphological tool for proving immunological mechanisms involved. In MCD there is no IF staining or low level of mesangial staining of IgM or C3.^6^ In FSGS, there is IgM, C3 granular pattern on IF staining.^10^ In MN, there is usually positive staining of IgG and C3 in granular patterns.^11^ In MPGN, IF was positive for C3, IgG and IgM.^12^

In the present work, 51.9% were male. This male predominance was very similar to what has been found by other local^13^ and international researchers.^14^,^15^ This could be attributed to increased susceptibility of males to glomerular diseases. We noted that 58.7% children were above 10 years of age. “A National cross-sectional survey” done by Nie S et al from China^15^ noted that adolescents formed the majority children having glomerular diseases. This could be because of better understanding and acceptance of kidney biopsies by younger children and their parents/guardians.

FSGS was found to be the commonest histopathology finding noted in 40.8% followed by MesPGN 19.1% and Membranous nephropathy 10.9%. Spectrum of GN has been found varying across different parts of the world. From Croatia, Batanic et al.^16^ noted very similar findings where they noted FSGS to be among 24.6% GN cases followed by MesPGN in 19.2%. Many researches from the United States.^17^ From India, it has been found that MesPGN was the commonest finding.^18^ A study from Bangladesh also found FSGS to be the commonest histological finding among children with GN.^19^ From Pakistan, MCD followed by FSGS to be the most common histopathological findings in GN.^20^ All these studies depict that spectrum of GN among children has variations among different geographies and regions and it is continuously evolving. These variations could be because of differences subjected to biopsy indications as well as patients referrals and racial predispositions among different nephropathies.^14^ No standard guideline exists regarding kidney biopsies among pediatric population.

IF embedded renal biopsies have been described as of “great diagnostic utility”.^7^ Local data are lacking but Alam AB et al from Bangladesh^19^ in their five years analysis found 86% of the patients to show positive immunofluorescence findings while IgM was the most predominant immune deposits among various glomerular diseases which is quite similar to what was found in the present research. Alam AB et al.^19^ also noted that among cases having FSGS, IF findings showed that 25.9% were IgG positive. We noted 41.7% of the cases with FSGS to be IgG positive while 53.7% were IgM positive in terms of IF findings. Similar to our findings, Nasir H et al.^9^ in a past local research found non-immune trapping of IgM in 40% cases. It was endorsed that the diagnosis of some glomerular diseases like IgAN and IgMN is based on IF examination.^9^

Comparatively big sample size is one of the strengths of this study. Comprehensive histological examination along with management and outcome with the help of follow ups are some of the other dimension which has not been studied previously in Pakistan. Selection bias among the study participants could be one of the main limitations of this study, as is usually the case among biopsy base researchers. We also could not analyze any information regarding previous treatment of the participants.

## CONCLUSION

Immunofluorescence proved an important diagnostic tool in reaching the exact diagnosis in various types of GN. Distinct correlation between IF findings and clinical course of various types of GN was observed. IF negative cases had better outcome and was not having progressive course of disease so prognosis remained better than IF positive cases in this study.

### Authors’ Contribution:

**FZ:** Conceived, Supervision, Proof Reading and responsible for the integrity of the study.

**MFM:** Data Analysis, Drafting.

**AAK:** Literature search, Discussion.

**RSS:** Data Interpretation, Discussion.
